# Precision and Practicality: A Novel Cellulose Gum-Based Indirect Bonding Protocol for Orthodontic Bracket Placement

**DOI:** 10.7759/cureus.96818

**Published:** 2025-11-14

**Authors:** Yugandhar Garlapati, B Rama Mohan Reddy, K N Anand Kumar, Siva Krishna Polisetty, C Venkata Reddy, Poornima Nayak, Sampath Krishna Veni, Joyasree Chatterjee, Seema Gupta

**Affiliations:** 1 Department of Orthodontics and Dentofacial Orthopedics, Government Dental College and Hospital, Kadapa, IND; 2 Department of Orthodontics and Dentofacial Orthopedics, Kothiwal Dental College and Research Centre, Moradabad, IND

**Keywords:** bonding, brackets, indirect, innovation, orthodontic, technique

## Abstract

Introduction: This study presents an innovative indirect bonding (IDB) protocol that utilizes a cellulose gum-based denture adhesive for precise orthodontic bracket placement. The aim of this study was to evaluate the efficacy of a cost-effective IDB protocol using a cellulose gum-based denture adhesive as a temporary cast adherent. The objectives included assessing bracket transfer accuracy via digital superimposition, laboratory efficiency, and adhesive remnant index (ARI) scores.

Materials and methods: This prospective study was conducted at the Department of Orthodontics, Government Dental College and Hospital, Kadapa, India, with ethical approval and informed consent. Working casts were prepared, and McLaughlin, Bennett, Trevisi (MBT) prescription brackets (n = 20) were aligned using pencil markings and an MBT height gauge. Dentofit R adhesive (ICPA Health Products Ltd.) was applied to bracket bases, activated by a three-second water mist, and stabilized for five minutes. Light-body polyvinyl siloxane (Zhermack Elite HD+) was used for gingival block-out, followed by thermoforming 1.0-mm soft thermoplastic sheets (Leone S.p.A.) using a vacuum/pressure thermoformer. Trays were immersed in water for five minutes to dissolve the adhesive, and the brackets were cleaned for intraoral bonding. Transfer accuracy was assessed by digitizing casts (T1) and intraoral arches (T2) using an intraoral scanner (Runyes R), with deviations measured using the MeshLab software at four bracket wing points. The laboratory cleanup time, bracket transfer accuracy, and ARI scores were evaluated. Statistical analyses were performed using paired t-tests, analysis of variance (ANOVA; one-way, two-way, and three-way), and Wilcoxon signed-rank tests (p < 0.05).

Results: Cervical wings showed greater mean deviation (0.45 ± 0.53 mm) than incisal wings (0.24 ± 0.18 mm) (p = 0.02), with no significant differences in horizontal versus vertical deviations (p = 0.172) or mesial versus distal sides (p = 0.766). The overall deviation was clinically acceptable (p = 0.333; mean difference 0.04 mm from the 0.3 mm threshold). The median ARI score was 0, indicating the absence of adhesive remnants. The cleanup averaged 20 minutes per arch.

Conclusion: The cellulose gum-based IDB protocol offered precise, cost-effective bracket placement with efficient cleanup and is suitable for resource-limited settings, although cervical wing accuracy requires further optimization.

## Introduction

Orthodontic treatment relies heavily on precise bracket placement in order to achieve optimal tooth alignment and occlusion. Traditional direct bonding, where brackets are positioned and bonded intraorally, offers simplicity but is prone to errors due to limited visibility, patient movement, and prolonged chairside time, which can lead to clinician fatigue and inconsistent outcomes [1,2]. By contrast, indirect bonding (IDB) allows brackets to be prepositioned on a working cast in the laboratory before transferring to the patient's teeth via a custom tray [2]. This technique enhances accuracy, reduces clinical time, and minimizes patient discomfort, making it particularly advantageous in complex cases [2,3].

Despite these benefits, conventional IDB methods face significant challenges. Early approaches, such as those using unfilled methyl-methacrylate adhesives, often resulted in bracket base contamination during transfer, compromising bond strength and increasing failure rates [1]. Subsequent innovations included the "Sugar Daddy" technique employing caramel candy as a temporary adherent and a method that used sticky wax applied to warmed brackets [4]. More recently, Sondhi's protocol in 1999 utilized adhesive pre-coated brackets for efficient bonding [5]. However, these methods frequently involve lengthy laboratory procedures, potential mesh contamination from residual adhesives, and excess composite flash around gingival margins, necessitating extensive cleanup and increasing costs [4].

Bracket failure rates in IDB remain a concern, with clinical trials reporting rates of up to 2.5-5.7%, often attributed to inadequate base cleaning and material remnants [6,7]. Moreover, the need for specialized equipment or adhesives limits accessibility in resource-constrained settings. To address these limitations, there is growing interest in low-cost, contamination-resistant alternatives that maintain transfer accuracy while streamlining workflows.

This study introduced a novel, economical IDB protocol using cellulose-gum-based denture adhesive cream as a temporary cast adherent for orthodontic brackets. Activated by water for enhanced tackiness and easily dissolved for cleanup, this approach combined light-body polyvinyl siloxane (PVS) for gingival block-out and thermoformed trays to ensure precise transfer with minimal contamination. The primary objective was to evaluate the digital transfer accuracy of a cellulose-gum denture adhesive-based IDB protocol, using mean linear deviation from a clinically acceptable threshold as the primary outcome measure. Secondary outcomes included laboratory cleanup time and adhesive remnant index (ARI) scores to assess efficiency and bracket base cleanliness.

## Materials and methods

This study was designed as a prospective in vitro experimental investigation using an in vivo component to validate transfer accuracy. This research was conducted at the Department of Orthodontics, Government Dental College and Hospital (GDCH) in Kadapa, Andhra Pradesh, India, where all laboratory procedures and analyses were conducted from June 2025 to September 2025. The study was approved by the Institutional Ethics Committee of the GDCH, Kadapa (Protocol no. 34/UAI/GDCH/KDP-25), and written informed consent was obtained.

The sample size was estimated using G*Power software (version 3.1.9.7; Heinrich-Heine-Universität Düsseldorf, Düsseldorf, Germany), and an effect size of 0.08 was obtained from a previous study, which analyzed transfer accuracy of indirect bracket bonding at 96% [8]. A minimum sample size of 20 teeth was required to obtain 80% study power and a 5% alpha error. The formula used for sample size was a one-sample proportion as follows:

\begin{equation} n = \frac{Z_{1-\alpha/2}^2 \times p(1-p)}{E^2} \end{equation}

where:

n is the required sample size.

Z is the desired confidence level at 95%.

p is the estimated or known proportion [9].

E is the desired margin of error (effect size).

**Figure 1:**
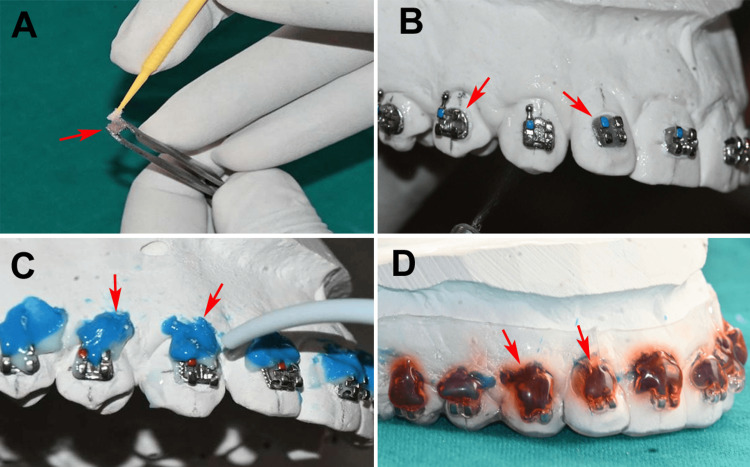
Steps of indirect bonding: (A) Dentofit R, a cellulose-gum-based denture adhesive cream was applied on the bracket base. (B) Brackets were then placed on the working casts, aligned to the reference markings, and gently pressed into position. (C) Application of a light-body polyvinyl siloxane material at the gingival surfaces of brackets. (D) Tray adhesive was applied on the light-body material to promote adhesion with the thermoplastic sheet and then thermoformed. Original images of working casts of the patient from the study.

Working casts of both dental arches were prepared using standard orthodontic impression techniques, and orthodontic brackets following the McLaughlin, Bennett, Trevisi (MBT) prescription (3M Unitek, Monrovia, California, USA) were selected for the protocol demonstration. Alignment was facilitated using a pencil to mark long-axis lines and an MBT height gauge to ensure the precise vertical positioning of each tooth. The temporary cast adherent employed was Dentofit R, a cellulose-gum-based denture adhesive cream (ICPA Health Products Ltd., Mumbai, India), which was applied to the bracket bases via an applicator tip (Figure 1A).

Brackets were then placed on the working casts, aligned to the reference markings, and gently pressed into position (Figure 1B). Subsequently, a three-way syringe delivered a fine water mist spray for 3 s across the full arch to activate the adhesive, transforming its grainy consistency into a gel form for enhanced bond strength, followed by a five-minute stabilization period at room temperature. To prevent voids in the gingival regions during tray formation, light-body polyvinyl siloxane (PVS) impression material (Zhermack Elite HD+, Zhermack S.p.A., Veneto, Italy) was applied to the gingival surfaces of the brackets (Figure 1C). A compatible tray adhesive (Polyether Adhesive, 3M Unitek, Monrovia, California, USA) was then added to the light-body material to promote adhesion with the thermoplastic sheet (Figure 1D). A 1.0-mm soft thermoplastic sheet (Leone S.p.A., Florence, Italy) was thermoformed over the model using a vacuum/pressure thermoformer (Bio-Art Plast Vac P7 Plus, Bio-Art Equipamentos Odontológicos Ltd., São Paulo, Brazil), after which the tray margins were trimmed with scissors or a handpiece.

**Figure 2:**
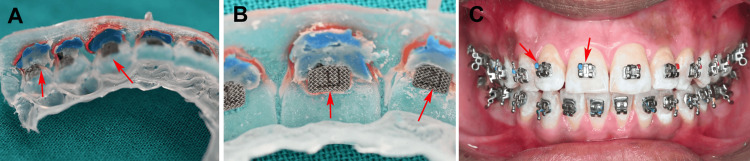
(A) Brackets were transferred to the thermoformed tray, (B) thermoformed tray showing brackets with adhesive, (C) full-arch indirect bonding. Original images from the study.

The tray containing the brackets was immersed in a bowl of water for five minutes to dissolve the denture adhesive, allowing easy removal from the model, and the bracket bases were cleaned under pressure with water from the three-way syringe, preparing them for intraoral bonding on the volunteer (Figure 2).

**Figure 3:**
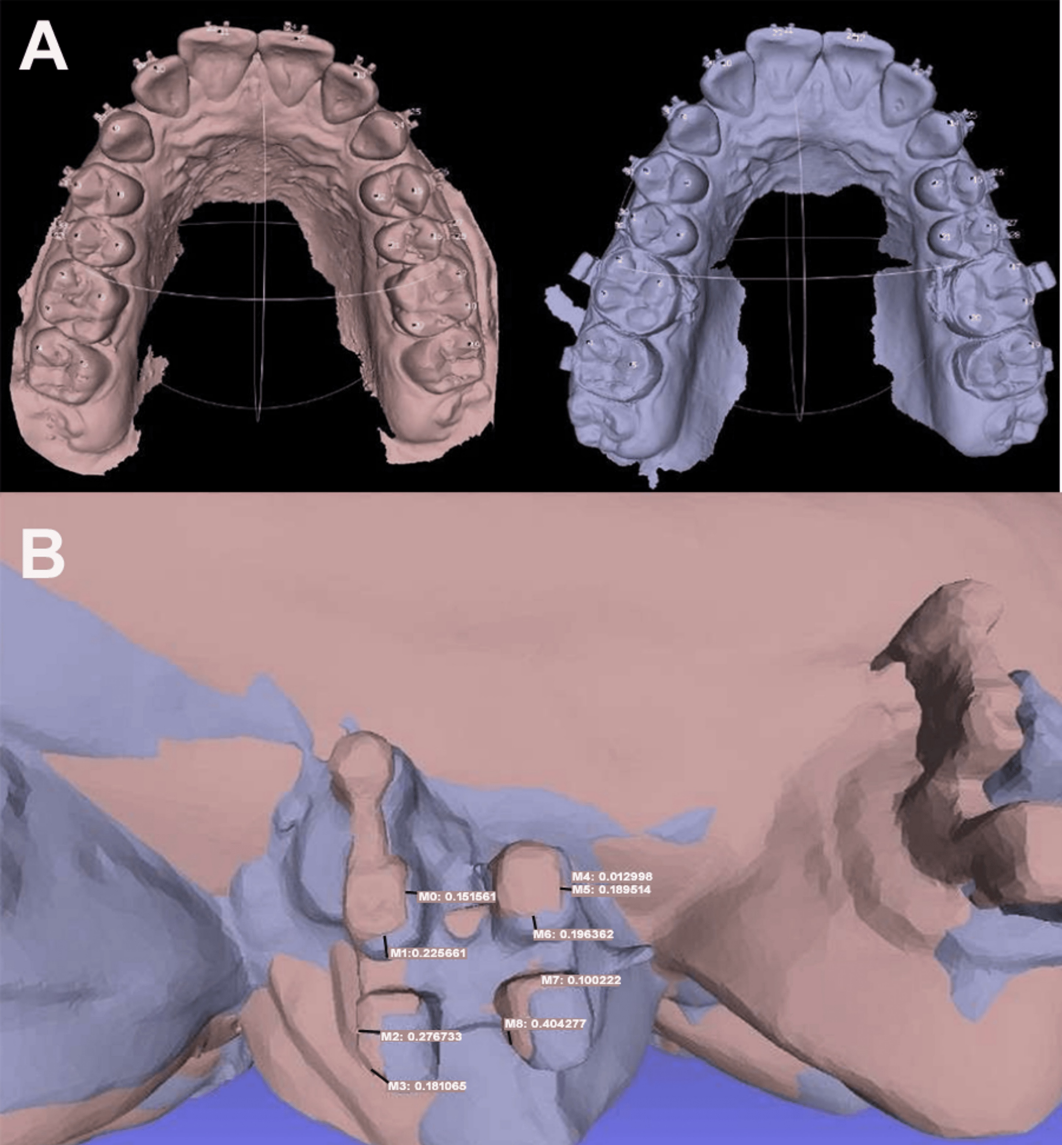
Digital superimposition and measurement of bracket transfer deviations: (A) Digital scan of the working cast (pink) superimposed with the intraoral scan of the patient (blue) using MeshLab software. (B) Measurement of horizontal and vertical deviations at four reference points (cervical and incisal bracket wings) on the superimposed models. The color-coded overlay illustrates linear discrepancies between intended and achieved bracket positions used to quantify transfer accuracy in the indirect bonding protocol. Original images from the study.

The transfer accuracy was evaluated by digitizing the bonded working models at time point T1 and the intraoral arches with brackets at T2 using an intraoral scanner (Runyes R, Runyes Medical Instrument Co., Ltd., Ningbo, China), followed by quantification of linear horizontal and vertical discrepancies in millimeters through digital superimposition (MeshLab software, Visual Computing Lab, Pisa, Italy), which was first calibrated by overlaying duplicate scans of the same model. Deviations were measured at four reference points per bracket, including the cervical distal wing, cervical mesial wing, incisal distal wing, and incisal mesial wing, across a sample of 20 teeth (Figure 3).

The laboratory cleanup time for the bracket meshes of each full arch was recorded using a stopwatch, averaging 20 minutes. Bracket meshes were inspected for surface contaminants post-cleanup through visual examination and scanning electron microscopy to confirm intact structures without remnants. ARI scores were assigned based on the amount of residual adhesive on the bracket bases, with scores ranging from 0 for no adhesive to 3 for all adhesives remaining with a distinct bracket impression [10].

Statistical analyses were conducted using IBM SPSS Statistics for Windows, Version 23.0 (released 2015, IBM Corp., Armonk, NY). The data from horizontal and vertical bracket deviation in four directions and adhesive remnant score were checked for normal distribution using the Shapiro-Wilk test. A normal distribution was found for bracket deviation; however, the ARI score was non-normal. A paired t-test was used to compare the horizontal and vertical deviations between the working cast (T1) and intraoral (T2) scans. One-way analysis of variance (ANOVA) was used to evaluate the differences between bracket positions for both horizontal and vertical deviations. Two-way ANOVA was applied to assess the interactions between wing type (cervical vs. incisal) and deviation type (horizontal vs. vertical) or wing side (distal vs. mesial), while three-way ANOVA was used to further explore the interaction effects of wing type, deviation type, and wing side. The Wilcoxon signed-rank test was used to assess differences in ARI scores, which exhibited a non-normal distribution. All tests used a two-tailed significance level of p < 0.05, with 95% confidence intervals (CI) reported for means, and effect sizes (Cohen's d) were calculated for significant findings.

## Results

The analysis revealed a statistically significant difference in transfer accuracy based on wing type, with cervical wings demonstrating significantly greater mean deviation (0.45 ± 0.53 mm) compared to incisal wings (0.24 ± 0.18 mm) (p = 0.02). In contrast, no significant differences were found between the horizontal and vertical deviation directions (p = 0.172), or between the mesial and distal sides of the brackets (p = 0.766). The inference is that the IDB transfer protocol is less accurate at the cervical wings, identifying a specific area for clinical improvement while demonstrating consistent performance across different directions and sides of the bracket (Table 1).

**Table 1:** Comparison of overall deviation in mm within each parameter using paired t-test. Data are presented as mean and standard deviation (SD) at 95% confidence interval (CI) for 20 brackets (n = 20). *p < 0.05 denotes statistical significance using paired t-test.

Bracket parameter (mm)		95% CI for mean	Mean ± SD (mm)	T stats	p-value	Effect size
Wing type	Cervical	0.28-0.62	0.45 ± 0.53	2.38	0.02*	0.53
	Incisal	0.18-0.3	0.24 ± 0.18			
Deviation type	Horizontal	0.27-0.54	0.41 ± 0.43	1.38	0.172	0.31
	Vertical	0.16-0.4	0.28 ± 0.37			
Wing side	Distal	0.23-0.48	0.36 ± 0.39	0.3	0.766	0.07
	Mesial	0.2-0.46	0.33 ± 0.42			

Two-way ANOVA revealed no significant interaction effects between wing type and deviation direction (p = 0.894) or between wing type and wing side (p = 0.718). However, cervical wings consistently showed greater positional deviation than incisal wings across all measurements, with the highest mean deviation observed in cervical-horizontal placement (0.52 ± 0.56 mm) compared to incisal-vertical placement (0.18 ± 0.12 mm). This indicates that while the interaction between factors is not statistically significant, bracket transfer accuracy is predominantly influenced by wing location, with cervical regions being particularly vulnerable to placement errors, regardless of direction or side (Table 2).

**Table 2:** Two-way analysis of variance (ANOVA) to compare mean deviation. Data are presented as mean and standard deviation (SD) at 95% confidence interval (CI) for 20 brackets (n = 20). p > 0.05 denotes no statistical significance using two-way ANOVA test.

Wing type	Deviation type	95% CI for mean	Mean ± SD (mm)	F stats	p-value
Cervical	Horizontal	0.26-0.78	0.52 ± 0.56	0.02	0.894
	Vertical	0.15-0.62	0.38 ± 0.5		
Incisal	Horizontal	0.2-0.39	0.3 ± 0.2		
	Vertical	0.13-0.24	0.18 ± 0.12		
Cervical	Distal	0.2-0.69	0.45 ± 0.53	0.13	0.718
	Mesial	0.2-0.71	0.45 ± 0.54		
Incisal	Distal	0.2-0.34	0.27 ± 0.16		
	Mesial	0.12-0.3	0.21 ± 0.19		

Three-way ANOVA revealed no significant three-way interaction between wing type, deviation direction, and wing site (p = 0.307). Despite this, a clear pattern emerged in the mean deviations, with the highest inaccuracy observed for cervical wings subjected to horizontal deviation at the distal site (0.60 ± 0.62 mm). In contrast, the greatest precision was achieved with incisal wings under vertical deviation at the mesial site (0.15 ± 0.13 mm). The inference is that while no complex interaction drives the inaccuracies, the cervical-distal area remains the most challenging area for precise bracket transfer, particularly in the horizontal plane, highlighting a consistent clinical vulnerability (Table 3).

**Table 3:** Three-way ANOVA analysis for mean deviation comparison. Data are presented as mean and standard deviation (SD) at 95% confidence interval (CI) for 20 brackets (n = 20). p > 0.05 denotes no statistical significance using three-way ANOVA test.

Wing type	Deviation type	Wing site	95% CI for mean	Mean ± SD (mm)	F stats	p-value
Cervical	Horizontal	Distal	0.16-1.04	0.60 ± 0.62	1.06	0.307
		Mesial	0.07-0.8	0.43 ± 0.51		
	Vertical	Distal	0.01-0.57	0.29 ± 0.39		
		Mesial	0.04-0.9	0.47 ± 0.6		
Incisal	Horizontal	Distal	0.19-0.45	0.32 ± 0.18		
		Mesial	0.11-0.44	0.27 ± 0.23		
	Vertical	Distal	0.14-0.3	0.22 ± 0.11		
		Mesial	0.06-0.24	0.15 ± 0.13		

The one-sample t-test comparing overall bracket transfer deviation to the clinically acceptable threshold of 0.3 mm revealed no statistically significant difference (p = 0.333), with a negligible mean difference of 0.04 mm. This indicated that the overall transfer accuracy was clinically acceptable. When analyzed by individual parameters, except for cervical and incisal deviation, no deviation significantly exceeded the threshold. The inference was that the IDB protocol successfully achieved a clinically acceptable overall level of accuracy (Table 4).

**Table 4:** One-sample t-test to compare mean deviation to the threshold value (0.3 mm). CI: confidence interval, *p < 0.05 denotes statistical significance using one-sample t-test.

Parameter	T stats	p-value	Mean difference (mm)	95% Cl of the difference	
				Lower limit	Upper limit
Overall	0.97	0.333	0.04	-0.05	0.13
Horizontal	1.57	0.125	0.11	-0.03	0.24
Vertical	-0.3	0.763	-0.02	-0.14	0.1
Mesial	0.46	0.648	0.03	-0.1	0.16
Distal	0.93	0.36	0.06	-0.07	0.18
Cervical	1.78	0.042*	0.15	-0.02	0.32
Incisal	-2.19	0.035*	-0.06	-0.12	0

The median value for the ARI score for brackets was 0, indicating that the majority of brackets had an ARI score of 0. This suggests that, in most cases, no adhesive remained on the bracket mesh after cleaning (Table 5).

**Table 5:** Frequency distribution of adhesive remnant index (ARI) scores. Data are presented as frequency (n) and percentage, where n denotes the number of brackets.

ARI scores	Frequency (n)	Percentage (%)
0	13	65%
1	7	35%

## Discussion

The findings of this study highlight the viability of a cellulose gum-based denture adhesive as a temporary cast adherent in IDB protocols, demonstrating a reliable bracket transfer with targeted areas for refinement. The pronounced inaccuracy of cervical wings relative to incisal wings underscores a common challenge in IDB techniques, where gingival proximity may exacerbate positional errors. This disparity could stem from uneven tray seating pressure or incomplete gingival block-out with a light-body PVS, allowing minor shifts during transfer. Such mechanisms align with prior research indicating that vertical and occlusogingival deviations often exceed horizontal deviations owing to anatomical contours and material interactions [11,12]. For instance, a systematic review and meta-analysis of IDB studies reported higher vertical linear errors (mean 0.09 mm) compared to mesiodistal (0.08 mm) directions, which was attributed to tray misfit and adhesive thickness variations [11]. It was further concluded that silicone tray transfer led to greater accuracy than vacuum-formed or 3D-printed trays. Similarly, another systematic review and meta-analysis revealed that 3D-printed trays showed an error of 0.095 mm in the mesiodistal direction, 0.114 mm in the buccolingual direction, and 0.111 mm in the vertical direction, with biases toward gingival shifts potentially linked to seating challenges in posterior regions [13]. These patterns suggest that the gel transformation of the water-activated adhesive, while enhancing tackiness, might introduce slight fluidity at the cervical margins, if not fully stabilized, contributing to the observed vulnerabilities. Consequently, it has been suggested to enhance the spacing between the dental arches and impression tray through modified designs to optimize the accuracy of the fit and minimize vertical discrepancies [14].

Armstrong et al. [15] indicated that alterations of ≥0.25 mm for the upper central and lower incisor brackets and ≥0.5 mm for the other dentition would be deemed clinically relevant. Our study indicated that most of the deviations were lower than 0.2 mm and, therefore, not clinically relevant. While overall mean deviations were within clinically acceptable limits, localized cervical-distal deviations reached up to 0.60 mm and may hold clinical significance. The absence of significant differences between horizontal and vertical deviations or mesial and distal sides indicates consistent protocol performance across bracket orientations, implying that the method mitigates directional biases often seen in traditional IDB. This uniformity may result from precise initial alignment using MBT gauges and long-axis markings, coupled with the even distribution of the adhesive via the applicator tips. Comparative literature supports this, as a previous study on IDB vacuum-formed trays showed translational errors generally below 0.2 mm across directions, with no marked side-specific discrepancies, emphasizing the role of tray rigidity in maintaining symmetry [16]. Similar findings have been reported by Castilla et al. [17].

The cellulose gum-based denture adhesive used in this study offers distinct advantages over similar materials, such as resin-based adhesives or silicone-based adherents commonly employed in IDB protocols. Unlike resin-based adhesives, which often require mechanical removal and may leave remnants that compromise bracket mesh integrity [18], the water-activated cellulose-gum adhesive dissolves completely within five minutes of immersion, ensuring clean bracket bases with an ARI score of 0, as observed in this study. Compared to silicone-based materials, which can be costly and less adaptable to gingival contours [4,18,19], the cellulose-gum adhesive is economical and transforms into a tacky gel with a 3-sec water mist, enhancing the ease of application and positional stability. However, the reliance of the cellulose gum adhesive on precise water activation may pose a minor challenge in controlling gel consistency compared to pre-formulated resins, potentially affecting the initial tackiness if over-saturated. Overall, its cost-effectiveness, rapid dissolution, and compatibility with vacuum-formed trays make it a compelling alternative in resource-conscious orthodontic settings.

The results of the study further revealed the lack of interaction effects in multi-way ANOVA, which reinforces that inaccuracies are primarily localized to cervical areas rather than compounded by directional or sided factors, a finding echoed in meta-analyses, where subgroup analyses revealed negligible jaw or side influences on overall accuracy [11]. This consistency makes the protocol a robust alternative to more complex digital workflows, particularly in resource-limited settings.

Regarding bracket mesh integrity and cleanup, a predominant ARI score of 0 reflects excellent adhesive dissolution post-immersion, ensuring minimal remnants and preserving mesh structures without contamination. This outcome likely arises from the water-soluble nature of cellulose gum, which facilitates rapid cleanup (averaging 20 min per arch) compared to resin-based adherents that often require mechanical abrasion [4,18,19]. Previous literature corroborates this advantage, where easier removal with soluble materials was noted, reducing laboratory time and bracket damage risk [18,19]. The efficient cleanup not only enhances laboratory workflow but also minimizes cross-contamination, aligning with infection control standards in orthodontic practice.

Overall, the protocol's alignment with a clinically acceptable threshold (no significant deviation from 0.3 mm) validates its precision, which is comparable to that of established methods. An umbrella review of IDB techniques concluded that indirect approaches achieve accuracy on par with direct bonding, with pooled data supporting high repeatability in bracket positioning [20]. The innovation of this study lies in leveraging an economical, readily available denture adhesive, bypassing costly custom resins or digital fabrication, which can elevate the expenses of conventional IDB. Integrating simple activation via water mist and PVS block-out reduces voids and contamination, potentially shortening chairside time relative to direct techniques that demand intraoral adjustments.

The clinical implications of this protocol are substantial, offering orthodontists in underserved regions an accessible tool for precise bonding, which could improve treatment predictability and patient outcomes in fixed-appliance therapy. Its minimal material requirements and straightforward cleanup may lower barriers to adopting IDB, fostering broader use in public health settings, such as government dental hospitals. Moreover, the focus on cervical accuracy highlights opportunities for protocol tweaks, such as reinforced gingival reinforcements or alternative tray thicknesses, to further improve the performance.

Limitations include the small sample size of 20 teeth from a single volunteer, which limits generalizability across diverse dentitions or malocclusions. The predominantly in vitro design, with limited in vivo validation, may overlook intraoral variables, such as saliva or patient movement. Reliance on specific brands restricts applicability, and the absence of long-term bond failure assessments overlooks the durability. Future studies should expand on multicenter trials with larger cohorts and longitudinal tracking to address these gaps. These findings, although encouraging, should be interpreted with caution given the single-operator and single-volunteer design. The variability in cervical-distal deviations underscores the need for multi-operator, multicentre studies with long-term bond-failure assessment to confirm reproducibility and clinical reliability, consistent with conclusions from recent umbrella reviews on IDB efficiency and limitations [20].

## Conclusions

This study validated the efficacy of a novel IDB protocol using cellulose gum-based denture adhesives for orthodontic bracket placement, demonstrating clinically acceptable transfer accuracy and minimal adhesive remnants. The protocol ensured efficient laboratory cleanup and preserved bracket mesh integrity, offering a cost-effective alternative to the traditional methods. Despite greater positional deviations at the cervical wings, the approach maintained consistent performance across directions and sides, thus enhancing its potential for widespread clinical adoption. This method streamlines orthodontic workflows, particularly in resource-limited settings, and highlights opportunities for further refinement to address cervical inaccuracies, promising improved accessibility and precision in orthodontic practice.
